# Triacetyluridine treats epileptic encephalopathy from *CAD* mutations: a case report and review

**DOI:** 10.1002/acn3.51257

**Published:** 2020-11-29

**Authors:** Aliya Frederick, Kimberly Sherer, Linda Nguyen, Shawn Ali, Anupam Garg, Richard Haas, Michelle Sahagian, Jonathan Bui

**Affiliations:** ^1^ Department of Neurosciences University of California San Diego San Diego California USA; ^2^ Rady Children’s Hospital San Diego California USA; ^3^ Department of Pediatrics University of California San Diego San Diego California USA; ^4^ University of California San Diego School of Medicine San Diego California USA

## Abstract

Refractory epilepsy and encephalopathy are frequently encountered in patients with inborn errors of metabolism. We report a case of an 8‐year‐old girl with history of developmental delay, autism and intractable epilepsy that was found to have a pathogenic variant in CAD. We briefly review the biochemical pathway of CAD and the preclinical and clinical studies that suggest uridine supplementation can rescue the CAD deficiency phenotypes. Our case demonstrates a relatively late‐onset case of refractory epilepsy with a rapid response to treatment using the uridine pro‐drug triacetyluridine (TAU), the FDA‐approved treatment for hereditary orotic aciduria.

## Case Presentation

An 8‐year‐old girl with developmental delay, autism, and intractable epilepsy presented with increased seizures in the setting of parainfluenza respiratory infection. She had a history of multiple seizure semiologies including tonic, generalized tonic–clonic, staring with behavioral arrest, and focal motor seizures. Seizures varied in frequency and duration, and she had several prior episodes of status epilepticus requiring hospitalization. Six months prior to presentation, she had prolonged status epilepticus, followed by regression in functional skills, gait ataxia, hand tremors, incoordination, and increased seizures.

She was born by normal vaginal birth. It was not clear when developmental delays were first noticed, but she was diagnosed with autism at age 4. Seizures started with febrile illness at 17 months old. Over time, seizures became increasingly difficult to control requiring escalating doses and number of anti‐convulsant medications. The family history showed childhood epilepsy and behavioral concerns on the paternal side. The parents were consanguineous being distant cousins.

At the time of admission, the patient was taking phenytoin 5 mg/kg per day, levetiracetam 68 mg/kg per day, and clobazam 1.5 mg/kg per day.

Video EEG was completed to characterize limb and eye movements concerning for seizures. Focal seizures were captured although the majority of episodes of concern were not seizures. The EEG was notable for diffuse delta slowing and multifocal epileptiform discharges, consistent with epileptic encephalopathy.

Complete blood count showed macrocytic anemia (Hgb 10.6 g/dL; MCV 91.4 fL) and mild thrombocytopenia (135 TH/*µ*L). Basic chemistries showed mild transaminitis.

Computed tomography imaging of the brain showed no acute abnormalities. Brain magnetic resonance imaging at age 4 years was normal. Her karyotype was normal. A chromosomal microarray showed areas of homozygosity without any clinically significant copy number variants. A commercial epilepsy gene panel testing 181 genes in 2019 was unremarkable. Her plasma amino acids, lactate, ammonia, pyruvate, lead, and urine organic acids were normal.

During the hospitalization, the patient developed worsening coordination, nystagmus, tremor, and encephalopathy initially thought to be secondary to medications. It became difficult to find an acceptable balance between seizures and medication side effects. An epilepsy gene panel testing 193 genes revealed a homozygous variant (c.98T > G. p.Met33Arg) in the Carbamoyl‐Phosphate Synthetase 2, Aspartate Transcarbamylase, and Dihydroorotase (*CAD*) gene, which was reported as likely pathogenic. This variant is found in the general population at very low frequencies in the heterozygous state, and *CAD* missense variants are depleted in the population databases, suggesting that mutations in this gene are pathogenic (https://gnomad.broadinstitute.org). The mutation lies within one of the areas of homozygosity that was noted on the patient’s microarray; however the variant was not confirmed in the parents as they declined genetic testing.

Pathologic variants in the *CAD* gene result in a rare neurometabolic disorder characterized by autosomal recessive early epileptic encephalopathy, developmental delay, and intractable epilepsy, as well as a macrocytic anemia.^1^ In its untreated form, this disorder is progressive and fatal, however, there are reports in the literature of patients who have been successfully treated with uridine supplementation.^1^, ^2^

## *CAD* Gene and Uridine Supplementation

The *CAD* gene, located on chromosome 2 (2p23.3), encodes a highly conserved trifunctional enzyme complex involving carbamoyl‐phosphate synthetase 2, aspartate transcarbamylase and dihydroorotase.^3^ This enzyme complex is important in the pathway for de novo synthesis of pyrimidine nucleotides. The final product, uridine monophosphate (UMP), is the substrate for all cellular pyrimidines and therefore essential for RNA and DNA synthesis. UMP derivatives (tri‐ and diphosphate, UTP/UDP) are also important in protein glycosylation, polysaccharide biosynthesis, and lipid metabolism. The importance of uridine is highlighted in the inborn error of metabolism disorder hereditary orotic aciduria (UMPS deficiency), in which there is a primary deficiency in uridine synthesis, leading to failure to thrive, megaloblastic anemia, congenital malformations, immunodeficiency, and developmental delay.^4^

Given that cells are able to take up and phosphorylate exogenous uridine, supplementation with uridine can provide a mechanism to overcome deficiencies in the synthetic pathway. Accordingly, uridine triacetate (or triacetyluridine, TAU) is the FDA‐approved treatment for hereditary orotic aciduria.^5^ Uridine therapy has also been used in uridine‐nucleotide depletion disease,^6^ for overdose of fluorouracil and capecitabine,^5^ and is currently under investigation for treatment of mitochondrial diseases. Supplementation with TAU is typically favored over uridine given the bioavailability of TAU is 4–6 times greater than uridine, thereby requiring lower dosages for treatment. TAU is absorbed in the gastrointestinal tract and readily converted to free uridine and acetate by esterases in the gut epithelium. Rare side effects include mild nausea, vomiting, or diarrhea.^5^

There is a growing body of evidence demonstrating that uridine supplementation can also be helpful in deficiencies occurring in earlier steps of pyrimidine synthesis, including *CAD* mutations. In the preclinical setting, Ng et al. (2015) demonstrated that fibroblast cells from a 4‐year‐old patient with compound heterozygous mutations in *CAD* expressed reduced de novo pyrimidine nucleotides and reduction in all UDP‐activated sugars tested.^7^ Supplementation of exogenous uridine rescued these abnormalities. In addition, a study using a CAD‐knockout cell line showed 16 of 25 biallelic variants, including our patient’s specific variant, failed to rescue or only partially rescued the growth phenotype of these cells in the absence of uridine.^8^ This suggests that these mutations diminish CAD activity and that uridine supplementation may be helpful to restore the normal phenotype.

In the clinical setting, Koch et al. described four patients with *CAD* mutations in 2017, two of which were siblings from a consanguineous family who harbored the same mutation as our patient.^1^ All four patients had developmental delay, followed by refractory epilepsy, epileptic encephalopathy, developmental regression at 2–4 years old, and anemia. Two of the four patients were treated with oral uridine 100 mg/kg per day divided in four dose administrations. These patients showed significant improvement in their clinical course. The treated child who had our patient’s mutation showed cessation of seizures for at least 6 months, with stable or improved gross motor, fine motor, cognitive and speech function. The anemia also normalized. Cultured fibroblasts from this patient initially showed reduced levels of UDP, UDP‐glucose, UDP‐*N*‐acetylglucosamine, CTP, and UTP, which all normalized with uridine supplementation. The other treated patient went from a minimally conscious state to being awake, alert, and able to take steps with assistance. The two patients that were not treated died at 4 and 5 years old after progressive neurodegeneration, loss of acquired skills, and refractory seizures. In 2020, a 5‐year‐old boy with a different compound heterozygous *CAD* mutation who shared a similar phenotype to those described previously also had significant improvement in his seizures, development, and anemia after uridine supplementation.^2^

## Case Conclusion

Prior to TAU therapy, our patient had up to 6–12 seizures daily, refractory to various combinations of phenytoin, levetiracetam, clobazam, cannabidiol, and carbamazepine, with encephalopathy and regression of functional skills. Supplementation with TAU was initiated at a dose of 100 mg/kg per day divided four times daily. After supplementation, seizures ceased within 4 days and her mental status improved. When assessed after 2 months, the patient was alert, playful, speaking in multi‐word sentences, had coordinated movements, and was ambulating independently. She was continued on clobazam 1 mg/kg per day, levetiracetam 40 mg/kg per day, and TAU. When last assessed, the patient had not had a seizure in 1 year since starting TAU supplementation and continued to make developmental progress.

## Discussion

Here, we present a child with an epileptic encephalopathy resulting from a homozygous variant in the *CAD* gene (c.98T > G.p.Met33Arg) whose seizures resolved and encephalopathy improved with TAU treatment. There is a growing body of work demonstrating that refractory epilepsy and encephalopathy are frequently encountered in patients with inborn errors of metabolism.^9^, ^10^ Furthermore, the effectiveness of therapies that are targeted to specific metabolic pathways has been well‐documented.^11^, ^12^, ^13^ For example, Hunt et al. (1954) documented a case of neonatal epilepsy resolved by daily pyridoxine treatment, with genetic studies in the past 2 decades highlighting the involvement of ALDH7A1 and PROSC in pyridoxine‐dependent epilepsy.^14^, ^15^ Since then, various metabolic pathways involving biotin recycling, glucose transport, creatine synthesis, purine metabolism, and Coenzyme Q10 deficiency have been implicated in pediatric epilepsy syndromes, with substantial response to replacement therapies.^9^ The success of uridine supplementation in the treatment of several cases of epileptic encephalopathy resulting from *CAD* mutations further demonstrates the impact of genetic‐based epilepsy therapies. Exome sequencing as well as targeted gene panels have been shown to play an increasingly important role in elucidating these rare and often *de novo* genetic variants underlying epileptic encephalopathies.^16^, ^17^

Our patient is the first child reported with intractable epilepsy and developmental regression secondary to *CAD* mutation who responded clinically to treatment with the uridine prodrug TAU. Exogenous uridine is thought to bypass the loss of *CAD* function, thereby rescuing pyrimidine synthesis and downstream pathways. Our case uniquely demonstrates a relatively late‐onset case of refractory epilepsy in the setting of a homozygous variant in the *CAD* gene, with a striking, rapid response to replacement therapy. As the number of reported cases of refractory epilepsy from inborn errors of metabolism continues to grow, it is critical for us to develop comprehensive, commercially‐available epilepsy gene panels for screening of this population to potentially prevent the long‐term detrimental effects of these metabolic pathway errors.

## Conflict of Interest

Aliya Frederick – reports no disclosures. Kimberly Sherer ‐ reports no disclosures. Linda Nguyen– reports no disclosures. Shawn Ali– reports no disclosures. Anupam Garg – reports no disclosures. Richard Haas– reports no disclosures. Michelle Sahagian ‐ reports no disclosures. Jonathan Bui‐ reports no disclosures.
